# Editorial: Current Research in Equid Herpesvirus Type-1 (EHV-1)

**DOI:** 10.3389/fvets.2019.00492

**Published:** 2020-01-10

**Authors:** Tracy Stokol, Gisela Soboll Hussey

**Affiliations:** ^1^Department of Population Medicine and Diagnostic Sciences, College of Veterinary Medicine, Cornell University, Ithaca, NY, United States; ^2^Department of Pathobiology and Diagnostic Investigation, College of Veterinary Medicine, Michigan State University, East Lansing, MI, United States

**Keywords:** equine, virus, equine herpes myeloencephalopathy (EHM), EHV, pathogenesis

Equine herpesvirus type-1 is a double stranded DNA virus found worldwide. It is a major pathogen of horses, causing infectious outbreaks and individual cases of abortion, equine herpesvirus myeloencephalopathy (EHM) and neonatal pneumonia (1). EHV-1 is costly to equine industries due to implementation of quarantine procedures and biosecurity measures and is a reportable disease in many US states. Due to its health and economic impacts, there is substantial ongoing research on EHV-1. This Research Topic highlights the breadth of studies being performed on EHV-1, ranging from a review of currently available *in vitro* models, basic research on molecular biology, studies on potential treatments for EHV-1 or its clinical sequelae, and descriptions of EHV-1-associated pathology after *in vivo* experimental infection.

Kamel et al. reviewed *in vitro* models for studying EHV-1 interactions with respiratory mucosal or endothelial cells. Although such models cannot replace *in vivo* experimental infections of horses or rodents, they allow testing of hypothesis under defined, controllable conditions. Since EHV-1 infects via the respiratory tract (1), blocking mucosal entry will essentially prevent infection. Thus, respiratory mucosal models, e.g., nasal explants or three-dimensional primary respiratory epithelial cell cultures, are important tools to understand and hopefully identify means to block infection. The current paradigm is that EHV-1 spreads systemically through a cell-associated viremia (1). Subsequent infection of placental and spinal vessels leads to the most serious clinical sequelae of infection; abortion and EHM. Although vascular bed-specific and hormone-modulated expression of adhesion molecules are likely involved in this tissue “targeting,” EHV-1 similarly infects endothelia in other sites (2). *In vitro* microfluidic models that permit real-time monitoring of leukocyte-endothelial interactions under physiological flow will be crucial in understanding how the virus infects endothelia and the role of associated virulence factors, such as the viral DNA polymerase polymorphism (3) and host tetraspanin 9 (4).

In molecular studies, Shakya et al. compared gene sequences, viral replication kinetics and *in vivo* pathogenicity of an attenuated KyA strain with virulent strains, RacL11 and Ab4. Despite more gene deletions, the KyA strain replicated efficiently in rabbit and human kidney, equine dermal and murine epithelial cell lines *in vitro* and in mouse lungs *in vivo*. KyA and Ab4 strains caused mild weight loss, whereas RacL11 was lethal, in CBA mice. It is likely that viral gene deletions or mutations will impair infectivity, replication, or latency in relevant equine cells, such as endothelial or respiratory epithelial cells. Thus, side-by-side strain comparisons in primary equine cells may help identify viral proteins or mutations that are important in viral pathogenesis.

There are few antiviral drugs that can be used for treating EHV-1 infection. Valacyclovir mildly reduces viral shedding and the degree of viremia in experimentally infected horses (5), however it is unknown if valacyclovir is efficacious in the field. Many viruses co-opt host cell components to facilitate replication. Using a lysine-specific demethylase-1 inhibitor, OG-L002, Tallmadge et al. tested whether host-associated histones are involved in viral replication *in vitro*. They found that OG-L002 reduced viral DNA loads and suppressed early viral gene production in equine fetal kidney cells, particularly when used with ganciclovir. However, neither drug inhibited viral replication in peripheral blood mononuclear cells *in vitro*, which is a useful but artificial model of cell-associated viremia. A multifaceted approach is likely needed to inhibit EHV-1 and targeting host proteins that are co-opted by the virus is an intriguing avenue that deserves further exploration.

Thrombosis occurs in vessels harboring EHV-1 antigen, causing hypoxic tissue injury and contributing to clinical symptoms associated with EHV-1. Stokol et al. tested whether heparin-based anticoagulants inhibit EHV-1-induced platelet activation *ex vivo*, using the latter test as a surrogate marker for the risk of EHV-1-induced thrombosis. In a double-blinded randomized cross-over trial, they found that low-molecular-weight heparin was more effective than unfractionated heparin at inhibiting viral-induced platelet activation. With concurrent *in vivo* evidence that experimentally infected horses are hypercoagulabe during the viremic phase of infection (6, 7), heparin anticoagulants may help reduce thrombosis. Indeed, heparin administration was associated with a reduced incidence of EHM in one clinical outbreak (8).

Holz et al. provided a detailed description of histopathologic findings after experimental intranasal infection of yearlings with wild type Ab4 and 2 modified Ab4 mutants (a N752 DNA polymerase polymorphism mutant and an EHV-4 gD glycoprotein replacement mutant, in which the gD of EHV-1 was replaced with that of EHV-4). Acute neurologic disease and necrotizing vasculitis in the spinal cord and eye was only seen in horses infected with wild type Ab4. However, mild vasculitis, ganglioneuritis, and chronic testicular inflammation were present in horses infected with wild type and mutant Ab4, with ocular lesions being seen in a higher proportion of the Ab4gD4 replacement mutant-infected horses. This data suggests that the viral mutants are reaching and infecting the vasculature of tissues affected by wild type EHV-1, despite not being associated with clinical neurologic disease in this cohort. The findings raise the possibility that clinical disease depends on rates of viral replication within endothelial cells, efficiency of viral transfer from the endothelium to extravascular tissue or immune cells, and localized endothelial and immune responses to the virus, particularly considering the link between tetraspanin 9 and EHM on genome-wide association scanning (4). Being difficult to study in the living horse, the use of aforementioned *in vitro* microfluidic techniques or generation of “body-on-the-chip” or three-dimensional models of the vascular bed-tissue microenvironment of the spinal cord and other tissues (9) would help to answer the many questions that remain regarding the pathogenesis of EHV-1-associated clinical disease.

In conclusion, this topic shows the continued progession of our knowledge on EHV-1. Further studies on host and viral interactions involved in viral entry, spread, and replication in the respiratory epithelium and specific vascular beds, including host immune responses or evasion of antiviral responses, are needed. Such studies are dependent on sophisticated *in vitro* and in *vivo* equine models and would be bolstered by the development of equine-specific reagents, e.g., monoclonal antibodies against cellular adhesion molecules, and information on virus crystal and antigenic epitopes, which is currently lacking.

## Author Contributions

The manuscript was written by TS and edited by GS.

### Conflict of Interest

The authors declare that the research was conducted in the absence of any commercial or financial relationships that could be construed as a potential conflict of interest.
